# Efficacy of Telerehabilitation for Ankylosing Spondylitis: A Case Report

**DOI:** 10.7759/cureus.95360

**Published:** 2025-10-24

**Authors:** Kensuke Nakamura, Ryosuke Tozawa

**Affiliations:** 1 Department of Rehabilitation, Podiatric Surgery and Medicine Ogikubo, Suginami, JPN; 2 Department of Physical Therapy, Faculty of Health Science, SBC Tokyo Medical University, Chiba, JPN

**Keywords:** ankylosing spondylitis, information and communication technology, physical exercise, physical therapy rehabilitation, telerehabilitation

## Abstract

Ankylosing spondylitis (AS) is a chronic inflammatory disease that causes significant pain and mobility limitations. In Japan, patients with AS often face difficulties accessing specialized rehabilitation due to physical restrictions and the uneven distribution of healthcare resources. Telerehabilitation, utilizing information and communication technology, has emerged as a promising alternative to address these barriers. This case study reports on the implementation of telerehabilitation for a patient with AS experiencing substantial daily life limitations, which resulted in improvements in pain, physical function, and disease activity. A woman in her 20s, diagnosed with AS in September 2024, experienced exacerbated lower back pain and arthralgia in multiple joints, leading to marked restrictions in daily activities and reduced exercise tolerance. Because of her difficulty commuting to medical facilities, remote exercise instruction via information and communication technology (ICT) was initiated in late October 2024 with the approval of her attending physician. The intervention consisted of a combination of chair and floor exercises focusing on spinal mobility and core stability. Exercise intensity was adjusted based on perceived exertion and pain. Four 60-minute sessions were conducted remotely using ICT, at intervals of 7-14 days. Pain was assessed using the numerical rating scale (NRS), daily physical function with the Japanese version of the Health Assessment Questionnaire (J-HAQ), and disease activity with the Bath Ankylosing Spondylitis Disease Activity Index (BASDAI). Evaluations were performed at baseline and after the fourth session. At the final evaluation, NRS scores decreased in six of the seven self-reported painful sites, with lower back pain improving markedly from 7 to 3. The mean J-HAQ score decreased from 1.75 to 1.50, and the BASDAI score decreased from 5.20 to 4.10. The patient also gained independence in "dressing and grooming." However, shoulder joint pain increased from an NRS score of 3 to 5, and the duration of morning stiffness lengthened. No notable adverse events were observed. This single-case study suggests that remote exercise instruction via ICT can improve pain, daily physical function, and disease activity in patients with AS who have limited access to traditional rehabilitation. Although challenges such as increased shoulder pain were noted, this case highlights the potential of telerehabilitation as a feasible and effective means of providing ongoing specialist guidance and enhancing quality of life for individuals with AS. Future research with larger cohorts and objective assessments is warranted to establish the efficacy and generalizability of these findings.

## Introduction

Ankylosing spondylitis (AS), a chronic immune-mediated inflammatory disease classified within the axial spondyloarthritis group, primarily affects males under 45 years of age [1]. In Japan, the reported prevalence is 2.6 per 100,000 individuals [2]. In 2015, AS was designated an intractable disease, making patients eligible for subsidies for medical expenses. The main symptoms of AS include lower back and buttock pain due to sacroiliitis and spondylitis, as well as pain in large peripheral joints, such as the hips, knees, and shoulders, which can significantly limit daily activities. As AS progresses, joint contracture and ankylosis of the spine and peripheral joints may occur, leading to severe mobility loss, restrictions in daily life, and even bedridden states [2]. Furthermore, reduced spinal mobility decreases chest wall expansion and can result in pulmonary complications, such as restrictive ventilatory impairment [3]. Given these challenges, early intervention by healthcare professionals is crucial to prevent reductions in joint range of motion and associated complications in patients with AS.

Several studies have reported the effectiveness of rehabilitation interventions for AS. A systematic review by Dagfinrud et al. demonstrated that group-based rehabilitation and aquatic exercise were more effective in improving spinal mobility and reducing pain compared with home-based exercise programs [4]. Similarly, Gurpinar et al. conducted a randomized controlled trial comparing physical therapist-supervised aquatic exercise, land-based exercise, and home-based exercise in patients with AS [5]. They found that supervised aquatic and land-based exercises significantly improved pain, spinal mobility, and functional capacity, whereas the home-based exercise group showed no significant improvements [5]. Gravaldi et al. also concluded in their systematic review that, although supervised physical therapy is effective, its benefits require maintenance through ongoing structured home exercise programs [6]. These studies highlight the limitations of patient-led home exercise programs and underscore the importance of continuous professional supervision and guidance. However, many patients with AS face barriers to accessing specialized rehabilitation services due to physical limitations, geographical constraints, and the uneven distribution of healthcare resources. In response to these challenges, telerehabilitation using information and communication technology (ICT) has emerged as a promising alternative [7,8]. Previous studies have reported that telerehabilitation provides improvements in physical function and quality of life compared to conventional in-clinic rehabilitation [8,9]. Despite these findings, evidence regarding the feasibility and effectiveness of telerehabilitation for AS in Japan remains limited.

The aim of this study is to present a detailed case of a Japanese patient with AS experiencing significant pain and restricted joint mobility, in whom telerehabilitation using ICT was implemented. By examining the outcomes of this intervention, we sought to explore the potential of telerehabilitation as a feasible and effective approach to overcoming access barriers and improving the quality of life in patients with AS.

## Case presentation

The participant in this study was a woman in her 20s. Her present illness began in early April 2024, with symptoms including low-grade fever, fatigue, and arthralgia of the shoulders, fingers, and knees. She initially consulted a local clinic, where she was diagnosed with suspected polymyalgia rheumatica and initiated on oral steroid therapy. However, by mid-May 2024, her symptoms had not improved, leading to her transfer to a university hospital for further evaluation. In September 2024, after tapering of the steroids prescribed by the previous physician, plain radiography and MRI were performed again. Abnormal findings were observed in the sacroiliac joints. Following specialist evaluation, Reiter’s syndrome and SAPHO (synovitis, acne, pustulosis, hyperostosis, osteitis) syndrome were ruled out, and the patient was diagnosed with AS. During steroid tapering, she experienced worsening lower back pain and exacerbated arthralgia in multiple joints. Methotrexate was initiated in early October 2024. By mid-October 2024, symptomatic improvement remained minimal, resulting in significant limitations in her daily life and a reduction in the frequency of her outings. The decreased frequency of leaving home led to reduced exercise tolerance, making it difficult for her to commute to medical facilities. Given this situation, the patient requested rehabilitation to alleviate her pain and improve her daily activities. Consequently, telerehabilitation using ICT was initiated in late October 2024, with approval from her attending physician.

This case report was conducted in accordance with the principles of the Declaration of Helsinki. The purpose and content of the study were explained to the participant both in writing and orally, and written informed consent was obtained. She was clearly informed of her right to withdraw consent at any time without disadvantage.

The intervention protocol was developed with reference to a study by Gurpinar et al. [5]. Telerehabilitation consisted of chair and floor exercises. Chair exercises are primarily focused on spinal rotation, lateral flexion, and hamstring stretching. Floor exercises included hip lifts, bird-dog poses, and cat-cow poses. Exercise intensity was guided to remain within the "somewhat easy" to "somewhat hard" range of perceived exertion, with repetitions and sets adjusted as needed according to pain levels. If compensatory or deviated movements were observed during exercise, immediate verbal instructions and demonstrative gestures were provided to prompt correction. The content of the intervention was standardized across all sessions, which lasted approximately 60 min, with adequate rest periods to prevent exacerbation of pain. Exercise instruction was provided remotely using the web conferencing service Zoom (Zoom Video Communications, Inc.). Sessions were held every 7-14 days, depending on the patient's physical condition, for a total of four sessions. Evaluation items included pain, daily physical function, and disease activity. Pain was assessed using the numerical rating scale (NRS). Daily physical function was evaluated using the Japanese version of the Health Assessment Questionnaire (J-HAQ) [10]. Disease activity was assessed using the Bath Ankylosing Spondylitis Disease Activity Index (BASDAI) [11]. Assessments were conducted twice: at baseline and after completion of the four telerehabilitation sessions.

**Figure 1:**
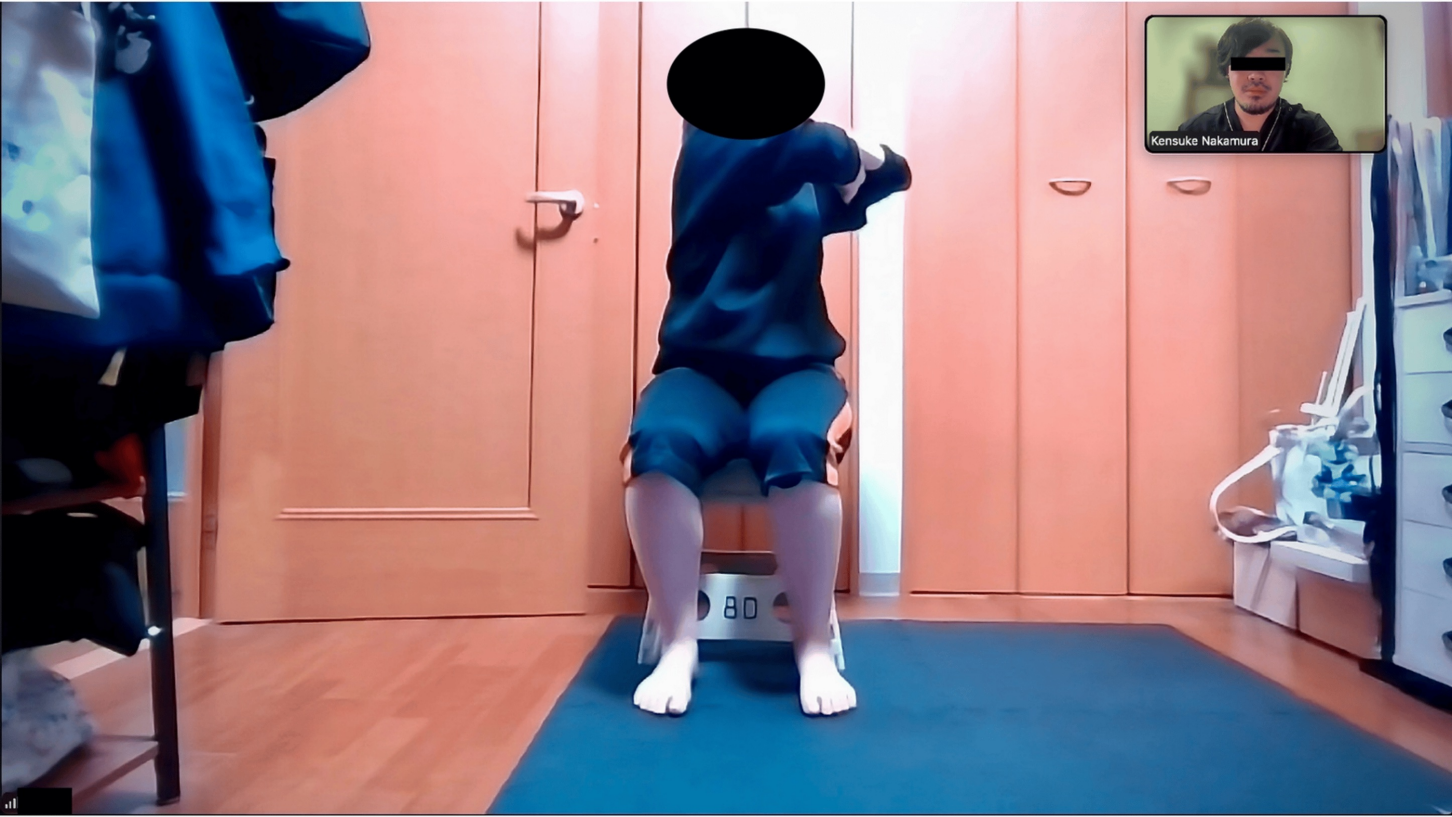
Implementation and conduct of synchronous telerehabilitation for ankylosing spondylitis in a home environment. The figure illustrates the visual arrangement for the real-time, synchronous tele-rehabilitation session.

Tables 1-3 present the baseline and final evaluation results for pain, J-HAQ, and BASDAI, respectively. Regarding pain assessment, a decrease in NRS scores was observed in six of the seven self-reported painful sites. Notably, lower back pain showed substantial improvement, decreasing from 7 to 3 on the NRS. However, shoulder joint pain increased from an NRS score of 3 to 5. The mean J-HAQ score decreased from 1.75 at baseline to 1.50 at the final evaluation (Table 2). For the J-HAQ category "Dressing and Grooming," which required assistance at baseline, the patient became able to perform the activity independently by the final evaluation. The BASDAI score improved from 5.20 at baseline to 4.10 at the final evaluation. While five of the six BASDAI categories showed decreased scores, the category "How long does your morning stiffness last from the time you wake up?" showed an increase. No notable adverse events were observed during the intervention period.

**Table 1:** Pain score trends before and after the intervention. NRS: Numerical rating scale

Body Part	Baseline Evaluation (NRS)	Final Evaluation (NRS)
Shoulder	3	5
Low Back	7	3
Hip	5	4
Knees	2	0
Ankle	3	0
Fingers	3	0
Toes	3	0

**Table 2:** Changes in the Japanese Health Assessment Questionnaire scores before and after exercise intervention.

Category	Baseline Score	Final Score
Dressing	2	1
Arising	1	1
Eating	2	1
Walking	1	1
Hygiene	2	2
Reach	2	2
Grip	2	2
Activities	2	2
Categories needing help	Dressing, Reach, Grip, Activities	Reach, Grip, Activities
Self-help tools	Bathtub chair, Bathtub grab bars	Bathtub chair, Bathtub grab bars
Total Mean J-HAQ Score	1.75	1.5

**Table 3:** Changes in disease activity (BASDAI) before and after exercise intervention. BASDAI: Bath Ankylosing Spondylitis Disease Activity Index

Category	Baseline Evaluation	Final Evaluation
1: How would you describe the overall level of fatigue/tiredness you have experienced?	8	6
2: How would you describe the overall level of neck, back, or hip pain you have had?	5	4
3: How would you describe the overall level of pain/swelling in joints other than your neck, back, hips?	4	3
4: How would you describe the overall level of discomfort you have from any areas tender to touch or pressure?	4	3
5: How would you describe the overall level of discomfort you have had from the time you wake up?	6	4
6: How long does your morning stiffness last from the time you wake up?(5 = 1 hour or more)	4	5

## Discussion

This case study reports the outcomes of a patient with AS who received four sessions of telerehabilitation using ICT. The findings suggest that telerehabilitation may be a viable option for patients who have difficulty accessing specialized rehabilitation because of physical limitations, geographical constraints, or the uneven distribution of medical resources.

In this case, the patient presented with significant limitations in daily life due to pain in multiple joints, including the lower back, prior to the intervention. Telerehabilitation led to marked improvement, particularly in lower back pain. This effect is likely attributable to exercises designed to improve spinal mobility and core stability, which were incorporated into the chair- and floor-based program. Reductions in J-HAQ and BASDAI scores were also observed, reflecting clinically meaningful improvements based on the minimum clinically important differences for AS [12,13]. Numerous studies have reported the effectiveness of exercise therapy and rehabilitation in patients with AS [5,6]. Most of these studies have involved direct rehabilitation by professionals such as physical therapists or supervised exercises [6,14]. Consistent with these findings, groups receiving direct professional guidance generally demonstrate greater improvements than those performing unsupervised home exercises [5,14]. Gravaldi et al. concluded that, although supervised physical therapy is effective, its benefits are difficult to maintain with home exercise programs alone [6]. This case reinforces the importance of continuous professional guidance for patients with AS. The telerehabilitation approach provided here is significant because it enabled ongoing professional input for patients with limited access to medical resources.

However, some negative outcomes were also observed. Shoulder joint pain increased from an NRS score of 3 to 5, and the BASDAI item on morning stiffness also worsened. The increase in shoulder pain may be explained by the exercise program’s primary focus on spinal and lower limb movements, with insufficient emphasis on shoulder-specific exercises. Soker et al. reported that nearly half of patients with AS (49.1%) had abnormal shoulder joint findings on MRI, underscoring that shoulder involvement is frequently overlooked and should be addressed early [15]. This highlights the need for targeted upper limb interventions alongside spinal exercises. The increase in morning stiffness could have been influenced by factors unrelated to the intervention, such as fluctuations in disease activity or sleep quality. Limitations inherent to remote rehabilitation, particularly the difficulty of performing detailed physical assessments and tailoring programs in real time, may also have contributed. These observations emphasize the need for refinement of remote rehabilitation approaches to better address the full spectrum of symptoms in AS.

Remote exercise instruction using ICT offers several advantages. Despite reduced exercise tolerance, which made it difficult for the patient to attend medical institutions, telerehabilitation reduced the burden of travel and enabled her to receive therapy. Performing exercises in a familiar home environment also lowered psychological barriers and supported the development of consistent exercise habits. To the best of our knowledge, only one prior study has investigated remote exercise interventions for AS. Acar et al. reported that eight weeks of thrice-weekly remote yoga group sessions improved disease activity and quality of life in patients with AS, suggesting that remote exercise may be both safe and effective [16]. Together with our case, these findings support the potential utility of telerehabilitation in this patient population. Nevertheless, several challenges remain. First, therapists cannot perform hands-on assessments or corrections during remote sessions, limiting the precision of movement guidance and the ability to evaluate muscle activity. Second, communication may be affected by video or audio disruptions. To address these issues, future studies should integrate objective assessments, such as wearable sensor technology, or consider hybrid models that combine remote and in-person sessions.

The limitations of this case study must also be acknowledged. The single-case design limits the generalizability of the findings. The intervention period of only four sessions was relatively short, and thus the sustainability of the observed benefits over the long term remains unclear. The evaluations relied primarily on patient-reported outcomes, underscoring the need to incorporate more objective measures of physical function in future research. Despite these limitations, this case provides valuable insights, suggesting that professional telerehabilitation using ICT can reduce pain and improve quality of life for patients with AS who face barriers to traditional rehabilitation. Future research should include multicenter collaborative studies with larger cohorts, interventions tailored to patients with varying disease activity, and randomized controlled trials with long-term follow-up to more robustly evaluate the efficacy and feasibility of telerehabilitation.

## Conclusions

This case study demonstrates that telerehabilitation delivered via ICT can improve pain, daily physical function, and disease activity in patients with AS who face limited access to conventional rehabilitation. Despite the short duration of the four-session intervention, significant reductions in pain, particularly lower back pain, were observed, alongside clinically meaningful improvements in J-HAQ and BASDAI scores. Although some challenges were noted, including increased shoulder pain and greater morning stiffness, the findings suggest that telerehabilitation is a feasible and effective approach to providing continuous professional guidance and enhancing the quality of life of patients with AS. Future studies involving larger cohorts, longer intervention periods, and objective outcome measures are warranted to confirm the efficacy and generalizability of these results.

## References

[1] Garcia-Montoya L, Gul H, Emery P (2018). Recent advances in ankylosing spondylitis: understanding the disease and management. F1000Res.

[2] Matsubara Y, Nakamura Y, Tamura N (2022). A nationwide questionnaire survey on the prevalence of ankylosing spondylitis and non-radiographic axial spondyloarthritis in Japan. Mod Rheumatol.

[3] Momeni M, Taylor N, Tehrani M (2011). Cardiopulmonary manifestations of ankylosing spondylitis. Int J Rheumatol.

[4] Dagfinrud H, Kvien TK, Hagen KB (2008). Physiotherapy interventions for ankylosing spondylitis. Cochrane Database Syst Rev.

[5] Gurpinar B, Ilcin N, Savci S, Akkoc N (2021). Do mobility exercises in different environments have different effects in ankylosing spondylitis?. Acta Reumatol Port.

[6] Gravaldi LP, Bonetti F, Lezzerini S, De Maio F (2022). Effectiveness of physiotherapy in patients with ankylosing spondylitis: a systematic review and meta-analysis. Healthcare (Basel).

[7] Yoshikawa K, Taima H (2022). [Fact-finding survey and report on the use of telerehabilitation around the world]. J Musculoskelet Phys Ther.

[8] Lin KH, Chen CH, Chen YY, Huang WT, Lai JS, Yu SM, Chang YJ (2014). Bidirectional and multi-user telerehabilitation system: clinical effect on balance, functional activity, and satisfaction in patients with chronic stroke living in long-term care facilities. Sensors (Basel).

[9] Odole AC, Ojo OD (2014). Is telephysiotherapy an option for improved quality of life in patients with osteoarthritis of the knee?. Int J Telemed Appl.

[10] Matsuda Y, Singh G, Yamanaka H (2003). Validation of a Japanese version of the Stanford health assessment questionnaire in 3,763 patients with rheumatoid arthritis. Arthritis Rheum.

[11] Garrett S, Jenkinson T, Kennedy LG, Whitelock H, Gaisford P, Calin A (1994). A new approach to defining disease status in ankylosing spondylitis: the Bath ankylosing spondylitis disease activity index. J Rheumatol.

[12] Kviatkovsky MJ, Ramiro S, Landewé R (2016). The minimum clinically important improvement and patient-acceptable symptom state in the BASDAI and BASFI for patients with ankylosing spondylitis. J Rheumatol.

[13] Soker G, Bozkirli ED, Soker E, Gulek B, Arslan M, Memis D, Yilmaz C (2016). Magnetic resonance imaging evaluation of shoulder joint in patients with early stage of ankylosing spondylitis: a case-control study. Diagn Interv Imaging.

[14] Barra L, Pope JE, Payne M (2009). Real-world anti-tumor necrosis factor treatment in rheumatoid arthritis, psoriatic arthritis, and ankylosing spondylitis: cost-effectiveness based on number needed to treat to improve health assessment questionnaire. J Rheumatol.

[15] Acar Y, Ilçin N, Sarı İ (2023). The effects of tele-yoga in ankylosing spondylitis patients: a randomized controlled trial. J Integr Complement Med.

[16] Liang H, Xu L, Tian X (2020). The comparative efficacy of supervised- versus home-based exercise programs in patients with ankylosing spondylitis: a meta-analysis. Medicine (Baltimore).

